# Implementation of the ‘Kimberley Mum’s Mood Scale’ across primary health care services in the Kimberley region of Western Australia: A mixed methods assessment

**DOI:** 10.1371/journal.pone.0273689

**Published:** 2022-09-02

**Authors:** Emma Carlin, Katherine Ferrari, Erica P. Spry, Melissa Williams, David Atkinson, Julia V. Marley

**Affiliations:** 1 The Rural Clinical School of Western Australia, The University of Western Australia, Broome, Western Australia, Australia; 2 Kimberley Aboriginal Medical Services, Broome, Western Australia, Australia; 3 Kimberley Population Health Unit, Western Australian Country Health Service, Broome, Western Australia, Australia; Waikato Institute of Technology, NEW ZEALAND

## Abstract

The Kimberley Mum’s Mood Scale (KMMS) was co-designed with Aboriginal women and healthcare professionals to improve culturally appropriate screening practices for perinatal depression and anxiety. This paper describes the implementation of the KMMS across the remote Kimberley region of Western Australia from January 2018 to December 2021. We used the Dynamic Sustainability Framework to progress the implementation and assess at the intervention, practice setting and ecological system level using a mixed methods approach to analyse implementation. Rates of administration and results of screening were described using a retrospective audit of electronic medical records. Analyses of KMMS training registry, stakeholder engagement and sustainability initiatives were descriptive. KMMS acceptability was assessed using qualitative descriptive approaches to analyse patient feedback forms (n = 39), healthcare professional surveys (n = 15) and qualitative interviews with healthcare professionals (n = 6). We found a significant increase in overall recorded perinatal screening (pre-implementation: 30.4% v Year 3: 46.5%, *P* < 0.001) and use of the KMMS (pre-implementation: 16.4% v Year 3: 46.4%, *P* < 0.001). There was improved fidelity in completing the KMMS (from 2.3% to 61.8%, *P* < 0.001), with 23.6% of women screened recorded as being at increased risk of depression and anxiety. Most healthcare professionals noted the high levels of perinatal mental health concerns, stress, and trauma that their patients experienced, and identified the KMMS as the most appropriate perinatal screening tool. Aboriginal women reported that it was important for clinics to ask about mood and feelings during the perinatal period, and that the KMMS was appropriate. Aboriginal women consistently reported that it was good to have someone to talk to. This study demonstrates that innovation in perinatal depression and anxiety screening for Aboriginal women is possible and can be implemented into routine clinical care with the support of a sustained multi-year investment and strong partnerships.

## Introduction

Perinatal mental health screening for high prevalence disorders of depression and/or anxiety is an established component of Australian clinical care [1]. Screening is typically undertaken by healthcare providers who administer the Edinburgh Postnatal Depression Scale (EPDS) [2]. It is well documented that Aboriginal Australian women are under-screened for perinatal depression and/or anxiety [3]. This is despite increased exposure to, and experience of, risk factors associated with perinatal depression and anxiety including poverty, intimate partner violence, adverse childhood experiences, and intergenerational trauma [4–8]. Reasons associated with the under-screening of Aboriginal Australian women are multifaceted. Some research suggests that Aboriginal women are presenting less to perinatal health appointments, which in turn reduces opportunity for clinical screening [3]. Other research suggests that Aboriginal Australian women may decline to participate in perinatal mental health screening due to the inappropriateness of the mainstream perinatal mental health screening tool (the EPDS) [9]. Fear of the consequences of disclosures, including removal of children and community stigma, have also been identified as a barriers to screening [10]. Additionally, it has been suggested that many Aboriginal Australian patients present with complex physical health conditions that require immediate attention, leaving limited clinical time for mental health screening or assessment [11]. These complex drivers of under-screening animate the need for health services to have the resources and commitment to ensuring Aboriginal women’s perinatal mental health screening is embedded within clinical models of perinatal care.

In the remote Kimberley region of northern Western Australia (WA) perinatal screening with the EPDS was recognised to be poor. The Kimberley Mum’s Mood Scale (KMMS) was developed in partnership between Aboriginal women and healthcare professionals as an alternative culturally appropriate approach to screening for perinatal depression and anxiety [9]. The KMMS is a two-part screening tool designed to be administered by a healthcare professional (e.g., General Practitioner, Midwife, Aboriginal Health Worker, Nurse). Part 1 of the KMMS uses local language and graphics in an adapted version of the EPDS. Part 2 of the KMMS is a ‘yarning’ [12] or narrative based assessment focusing on a woman’s risk and protective factors across seven psychosocial domains–support; stressors; self-esteem/anxiety; relationships; childhood experiences; drug and alcohol use; and social, emotional and cultural wellbeing [13]. The KMMS was validated in a clinical trial of 91 women, demonstrating the KMMS was clinically reliable and acceptable to women and their healthcare professionals [14].

A previous publication documented the KMMS pre-implementation planning and readiness work in the Kimberley region during 2017–2019 [11]. This demonstrated that many healthcare professionals were not using KMMS Part 2. Concerns over a lack of time and a perception this type of care was out of clinical scope were apparent [11]. Some healthcare professionals identified that the KMMS was only appropriate for women with literacy issues and stated they were not using the KMMS for Aboriginal women they deemed to be ‘literate’, further marginalising use of the KMMS [11]. In contrast, the Aboriginal women interviewed in the pre-implementation study considered the KMMS to be important for all Aboriginal women and placed high value on Aboriginal women having the time and space to ‘yarn’ with healthcare professionals about issues that are important to them [11]. The pre-implementation study identified a range of practical improvements to assist with the KMMS implementation. These included a KMMS graphic revision, significant further development of the training program and integration of the KMMS into the electronic medical record systems used by both Aboriginal Community Controlled Health Services (ACCHSs) and WA Country Health Service (WACHS) [11].

From 2019–2021, implementation of the KMMS has progressed across the primary healthcare providers in the Kimberley region. We have used the Dynamic Sustainability Framework [15] for planning the implementation and subsequent monitoring and evaluation of the KMMS. The Dynamic Sustainability Framework is an approach within the broader field of implementation science [16,17]. It is concerned with how an intervention, practice setting, and ecological system ‘fit’ together to support the implementation in an environment of ongoing change [15]. We have interpreted the domains of the Dynamic Sustainability Framework to refer to the KMMS as the intervention, the workforce and patient population as the practice setting, and the regional systems and structures that support primary healthcare delivery as the ecological system. This paper assesses the implementation process and outcomes of the KMMS across the Kimberley region using these modified domains of the Dynamic Sustainability Framework.

## Methods

### Research design

This study uses a mixed methods approach [18] to explore real-world outcomes of KMMS implementation in the Kimberley using the Dynamic Sustainability Framework [15]. This study aligns to the Standards for Reporting Implementation Studies (StaRI) Statement [17] and our statement against the standards is available (S1 Checklist).

The quantitative component of the study descriptively analysed a retrospective audit of the ACCHSs electronic medical records to explore rates of administration and outcomes of perinatal mental health screening via the KMMS. The data are reflective of two time periods. Time period 1 (T1) captures the pre-implementation phase (January–December 2018), Time period 2 (T2) captures the last year of the implementation study (January–December 2021). Rates of screening using the EPDS for Aboriginal women were also assessed at each time period to ensure a full assessment of screening practices and outcomes across the ACCHS.

Acceptability [19,20] of the KMMS was assessed from a healthcare professional and patient perspective using qualitative descriptive approaches [21]. We analysed patient feedback forms, healthcare professional surveys, and undertook qualitative interviews with healthcare professionals exploring their use of the KMMS. Analysis of KMMS training registry, and KMMS stakeholder interactions and engagement, alongside an assessment of partnership processes and KMMS sustainability initiatives were also described.

This project was endorsed by the Kimberley Aboriginal Health Planning Forum Research Subcommittee. It has approval from the Western Australian Aboriginal Health Ethics Committee (Project 781) and WACHS Human Research Ethics Committee (RGS 206). The project is aligned with the Australian National Health and Medical Research Council’s guidelines for ethical conduct in Aboriginal and Torres Strait Islander Health Research [22].

### Kimberley region

The Kimberley region is one of the most sparsely populated regions of Australia [23]. The population is estimated at approximately 34 000 people, with 42% of the population identifying as Aboriginal [24]. The population lives in remote communities, several small towns, and one medium-sized town. Health care is provided by ACCHSs and WACHS, a State Government health service. Aboriginal people in the Kimberley demonstrate resiliency and survival despite the intergenerational impacts of colonisation, which contribute to high levels of socioeconomic disadvantage, high burden of chronic diseases, and elevated levels of psychological distress [25,26].

The reported number of Aboriginal and/or Torres Strait Islander women residing in the Kimberley region who presented for antenatal care and gave birth in 2018, 2019, and 2020 was 337, 365, and 381, respectively [27]. Most of these women would have received antenatal care from Kimberley ACCHSs and/or WACHS. This usually consists of a shared care arrangement between the ACCHSs, WACHS Community Health and WACHS hospitals depending on a woman’s location, and personal preference. Kimberley ACCHSs use an electronic medical record system (MMEx; ISA Technologies, Perth, Australia). WACHS community health use electronic records (prior to 2018 Communicare (Telstra) and after 2018 Community Health Information System (CHIS; Telstra)). WACHS hospital based antenatal care continues to predominantly use paper-based records.

### Participants

A total of 137 healthcare professionals attended KMMS administrator training during the implementation period (2019–2021), all were followed up with an acceptability survey. The survey was emailed via a Survey Monkey^®^ link in November 2020 and again in May 2021 with a request for the healthcare professional to either complete the survey or participate in a qualitative interview. A total of 21 healthcare professionals participated in the acceptability study. Of these 15 participated via the online survey and six via a qualitative interview. Four interviews were conducted face to face, while two were conducted via video conference. All interviews were undertaken by KF and conducted during working hours at the participant’s place of work. Respondents participating in the interview provided written informed consent. Consent was implied for those healthcare professionals completing the anonymous health professional acceptability survey.

Healthcare professionals were asked to consent patients to the KMMS implementation study while using the KMMS as part of routine clinical care. Women who consented to be part of the study were asked to complete a short user acceptability survey. A total of 39 Aboriginal women consented to the implementation study. All women provided written informed consent permitting the use of their feedback in publications and reports. These forms were anonymous and are not linked to a woman’s KMMS assessment.

### Data analysis

#### Audit

All files were retrieved from the ACCHSs MMEx records. Reporting structures had been developed in parallel with WACHS in the establishment phase of the project. Despite ethical approval and consistent engagement with the WACHS Research Governance System, in part due to changing electronic medical record systems during the project, we were unable to access comparable automated KMMS data reports from WACHS.

**Fig 1:**
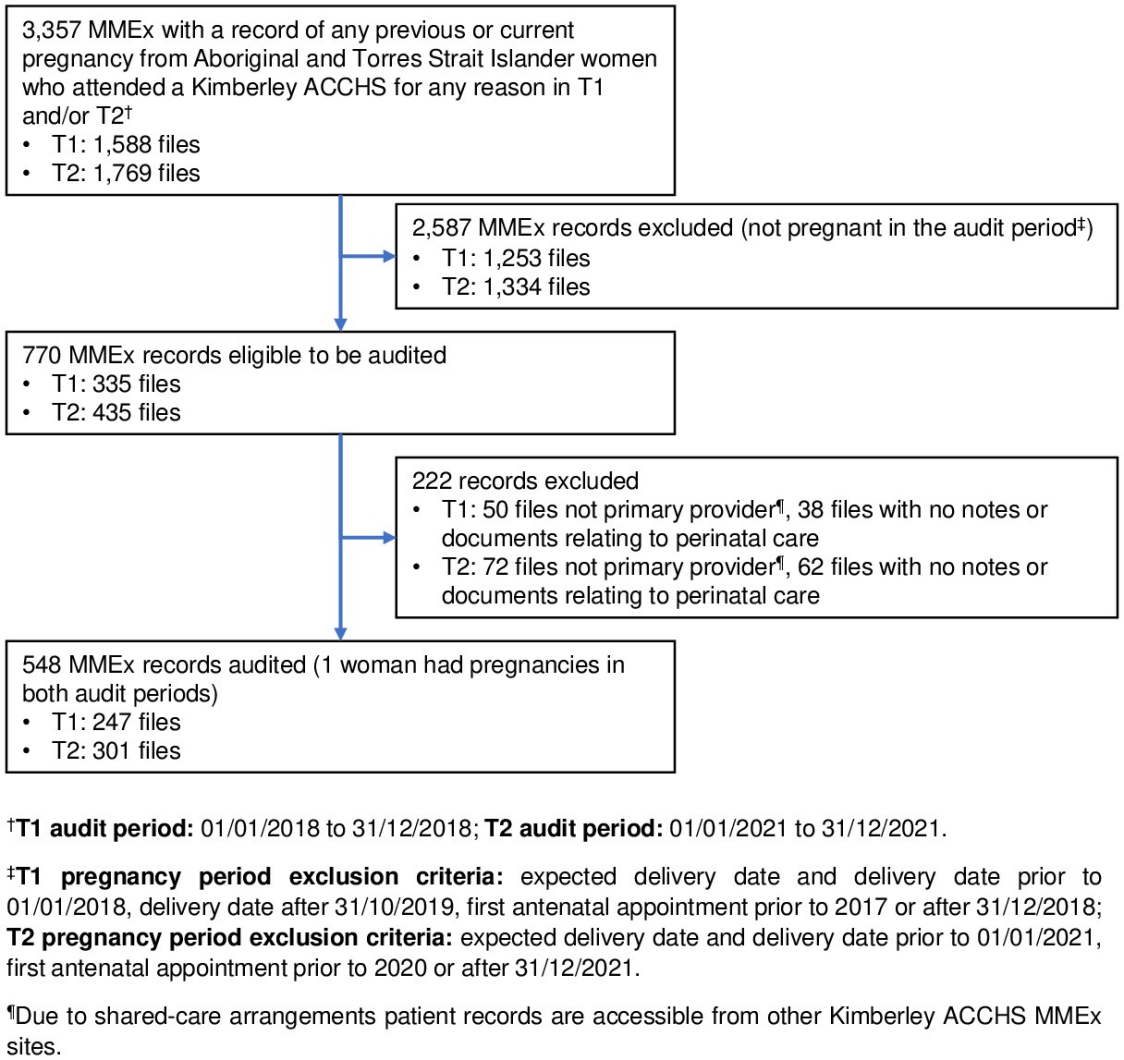
Flow chart for inclusion in the KMMS audit at timepoint 1 and timepoint 2.

Audit participants were Aboriginal women who were pregnant within either audit period (T1: 1/01/2018-31/12/2018; T2: 1/01/2021-31/12/2021) and received antenatal care from a Kimberley ACCHS for that pregnancy. Recorded pregnancy status, attendance and KMMS/EPDS use were obtained from each ACCHS MMEx site. Due to a limitation of MMEx batch exports, the initial MMEx export identified all Aboriginal and/or Torres Strait Islander women who had attended a Kimberley ACCHS on at least one occasion during T1 and/or T2 and had ever been recorded as pregnant (3357). Hence, most records (2809) were excluded as the pregnancy was outside the audit period or the Kimberley ACCHS MMEx site was not the primary provider for perinatal care (Fig 1).

After all exclusions, 547 MMEx records were audited for KMMS and/or EPDS use within each audit period. KMMS items recorded on MMEx were used to determine if the KMMS was administered with fidelity to the KMMS protocol [13]. Fidelity to the protocol requires a completed KMMS (Part 1, Part 2 and an overall risk assessment) recorded in the woman’s MMEx file. If the KMMS was ceased because the woman was identified as being at ‘immediate risk’ then the KMMS was also classified as completed.

Data were compiled in Microsoft Excel 365 (Microsoft), then imported into Stata 17 (StataCorp). Differences in characteristics between audit periods were compared using χ^2^ tests for categorical data and Mann–Whitney tests for continuous non-parametric data. *P* < 0.05 was defined as statistically significant.

#### Qualitative

Interviews with healthcare professionals were professionally transcribed into individual Microsoft Word documents. These were imported into NVivo 12 (QSR International), coded and analysed by authors EC and KF using a directed qualitative content analysis approach [28]. Steps in this approach included using our interview schedule (S1 File) as an initial tool for organising and categorising data. Data from the online surveys were also coded using categories established during the qualitative interview analysis, with additional codes created as required. A difference in the thickness of data between the in-depth interviews and the online surveys was apparent but considerable free text responses in the surveys permitted exploration of themes across the surveys and interviews. Use of surveys and interviews was chosen as a means of optimising healthcare professional participation in the acceptability study. After the data was initially coded, we evaluated our coding framework, recorded instance of response similarities in the data, assessed for divergencies, and assessed for emerging themes. Three themes emerged from the analysis process of the combined healthcare professional data: (1) KMMS: the ‘right’ approach’; (2) social determinants and amplified risk; and (3) screening as support. The final themes were reviewed by the research team (EC, KF, ES, DA and JM) and key quotes chosen.

User acceptability survey results from Aboriginal women were collated and descriptively analysed (EC, KF). Findings were then reviewed by a different author (ES) who is a Kimberley Aboriginal woman. The considerable free text responses to survey questions were analysed using a directed qualitative content analysis approach using the same steps described above for the coding and analysis of the healthcare professional’s data [28]. The final themes were reviewed by the research team (EC, KF, ES, DA and JM) and key quotes chosen. Cultural oversight of the data analysis was provided by ES. Inclusion of the free text responses from Aboriginal women in this manuscript ensures the voice of Aboriginal women is heard [29,30] while minimising the research burden of a more lengthy follow up qualitative interview.

## Results

### Domain 1: Intervention

As the implementation project progressed there was a significant increase in recorded overall perinatal screening (30.4% v 46.5%, P < 0.001) with a large increase in use of the KMMS (13.8% v 46.4%, P <0.001) and a significant decrease in screening with the EPDS (17.8% v 0.3%, P <0.001) (Table 1). There was also an increase in all items of KMMS (Part 1 and Part 2, and overall risk assessment or immediate concerns raised) being recorded (2.3% v 61.8%, P <0.001). In T2 there was no difference in KMMS Part 1 scores between women who had no overall risk assessment recorded and women who did have a risk assessment (median (range): 6 (0–25) v 8 (0–21), P = 0.57). Of the 140 women screened with the KMMS in T2, 23.6% had at least one record of having an elevated risk (immediate concerns or at moderate or high risk of depression and anxiety).

**Table 1:** Perinatal mental health screening across Kimberley ACCHS pre KMMS implementation project (T1) and in the final project year (T2).

	T1 (2018)	T2 (2021)
No. of women audited^¶^	247	302
Median age (IQR) at the start of each audit year	24.3 (20.8–28.9)	23.7 (20.3–29.3)
No. of women with EPDS and/or KMMS documented on MMEx^†^	75 (30.4%)	140 (46.4%)*
No. of women with EPDS recorded on MMEx	44 (17.8%)	1 (0.3%)*
1 EPDS	30 (12.2%)	1 (0.3%)
2 EPDS	12 (4.9%)	0
3 EPDS	2 (0.8%)	0
No. of women with KMMS recorded on MMEx	34 (13.8%)	140 (46.5%)*
1 KMMS	27 (10.9%)	104 (34.4%)
2 KMMS	4 (1.6%)	28 (9.3%)
3 KMMS	2 (0.8%)	6 (2.0%)
4 KMMS	0	2 (0.7%)
KMMS items recorded on MMEx		
KMMS with no KMMS document on file^‡^	8 (18.6%)	7 (3.8%)
KMMS with Part 1 only	30 (69.8%)	54 (29.0%)
KMMS with Part 1 & 2, but no overall risk	4 (9.3%)	10 (5.4%)
KMMS with Part 1 & 2, overall risk or immediate concerns^§^	1 (2.3%)	115 (61.8%)*
KMMS risk assessment recorded on MMEx		
No overall risk recorded	94 (79.1%)	65 (35.0%)
Low risk	8 (18.6%)	84 (45.2%)
Moderate risk	0 (0%)	31 (16.7%)
High risk	1 (2.3%)	3 (1.6%)
Immediate concerns raised	0 (0%)	3 (1.6%)
Highest KMMS risk recorded for each woman audited		
Not screened	172 (69.6%)	161 (53.5%)
Not recorded	67 (24.1%)	45 (14.9%)
Low risk	7 (2.8%)	63 (20.9%)
Elevated risk (moderate, high or immediate concerns)	1 (0.4%)	33 (11.0%)

ACCHS = Aboriginal Community Controlled Health Services. KMMS = Kimberley Mum’s Mood Scale. T1 = Timepoint 1. T2 = Timepoint 2. IQR = interquartile range. EPDS = Edinburgh Postnatal Depression Scale.

* *P* <0.001 using the Chi Squared test.

^¶^One woman was included in both time points.

^†^T1: 2 women had 1 EPDS and 1 KMMS recorded, 1 woman had 1 EPDS and 2 KMMS recorded; T3: 1 woman had 1 EPDS and 1 KMMS recorded.

^‡^Electronic medical records indicate that the KMMS was undertaken but no KMMS document or details could be found.

^§^KMMS protocol states that if a woman is at immediate risk, cease KMMS and follow organisational policy.

### Domain 2: Practice setting

Results presented under this section include the findings from the user acceptability studies relating to administering healthcare professionals and Aboriginal women who received the KMMS during routine clinical care.

#### Sample frame—Healthcare professionals

Responses to the online survey were anonymous and no demographic data was collected other than the respondent’s role. Of the 15 respondents, one identified as a General Practitioner (GP), four as Midwives, seven as Child Health Nurses and three as Remote Area Nurses. Of the six healthcare professionals who participated in a qualitative interview, respondents were asked basic workplace and demographics questions (Table 2).

**Table 2:** Characteristics of healthcare professionals interviewed for the KMMS implementation acceptability study.

Participant	Gender	Aboriginal	Time working in the Kimberley	Role	Provider
1	Female	No	Three years	Midwife	WACHS
2	Female	No	Four years	Remote Area Nurse / Midwife	ACCHS
3	Female	No	18 months	Child and Maternal Health Nurse	ACCHS
4	Female	No	Eight years	Clinical Midwife	ACCHS
5	Female	No	11 years	Maternal and Child Health Nurse	WACHS
6	Female	No	18 months	Midwife	WACHS

WACHS = WA Country Health Service. ACCHS = Aboriginal Community Controlled Health Service.

#### KMMS: The ‘right’ approach

The majority of respondents identified the KMMS as the most appropriate perinatal screening tool for Aboriginal women across the Kimberley. Healthcare professionals routinely described the KMMS as ‘easy to use’, ‘practical’, ‘resiliency focussed’ and ‘holistic’. Many respondents discussed the importance of introducing screening as a ‘universal’ component of clinical care to ensure the patient did not feel ‘singled out’. Respondents discussed how they introduced the KMMS as a tool to help all women get the right support from their healthcare professionals:

*You can’t provide good care unless you know her well and what her life is like at home*. *And that is what I tell the women who are coming to see me*.

Interview respondent 006

All healthcare professionals spoke about how mental health problems can only be adequately assessed through exploration of a women’s story and through an understanding of her context. KMMS Part 2 was highly valued as a framework to guide these discussions.

*It’s* [depression/ anxiety] *often not to do with a score or number*. *What we need to look at is all the challenges and try and minimise those risk factors in their life that’s causing this stress and this depression*, *this can only happen with talking things through*.

Interview respondent 001

*We had one lady that scored high on Part 1*, *but that week her partner had been flown down to Perth for life-saving surgery*, *so that week she just felt totally stressed out and very overwhelmed and so she scored really*, *really high*, *but the week later*, *after he had his surgery and everything was totally fine she didn’t feel like that anymore*. *So you need to have that conversation in Part 2 to understand what’s going on with Part 1*.

Interview respondent 002

Two healthcare professionals participating in the online survey answered that they were ‘unsure’ if the KMMS was the right approach for Aboriginal women.

*Some Kimberley Aboriginal women are very well schooled and are able to complete an EPDS as well as any other person*.

Survey respondent 014.

*In my experience when the KMMS is used the outcome and follow up is enhanced*. *I do have some mums who decline or feel uncomfortable doing part B* [Part 2].

Survey respondent 001.

Two healthcare professionals raised similar themes within the in-depth interviews. Both suggested they were concerned that the language and graphics in KMMS Part 1 could be perceived as ‘dumbed down’. They both stated the value of KMMS came in Part 2.

*No*, *I have never actually asked women if they find it* [Part 1] *appropriate*, *but I know that they all appreciate the conversation* [Part 2] *anyway*. *They will say things like they feel cared for*, *like somebody actually cares how they’re going at home…*. *It is not just about depression and anxiety; it’s about the whole picture*, *what’s making them feel like that*. *So it is well-received the vast majority of the time*.’

Interview respondent 005

When asked who was facilitating KMMS screening in the clinic, interview respondents stated this was the role of Midwives, Child Health Nurses and occasionally Doctors. Some interview respondents stated there were no Aboriginal Health Workers attached to their individual Maternal and Child Health teams. Other respondents stated that if Aboriginal Health Workers were part of their team they were busy taking the initial observations of a patient and triaging them for further clinical care. It was suggested that in current clinic structures Aboriginal Health Workers did not have time, scope, or access to private offices; requirements that were seen as necessary for perinatal mental health screening. Most respondents felt the inclusion of adequately supported and resourced Aboriginal Health Workers specific to Maternal Child Health teams would be useful for enhancing culturally secure engagement and support of Aboriginal mothers during the perinatal period.

#### Social determinants and elevated perinatal mental health risk

Healthcare professionals identified that the Aboriginal women they cared for experienced high levels of perinatal mental health concerns, stress and trauma. Overcrowded and insecure housing, experiences of abuse (including domestic violence), exposure to problematic use of alcohol or drugs and current involvement with statutory child protection services were identified as common stressors that affect a woman’s mental health.

*Yes*, *it’s* [perinatal mental health] *huge*. *I’d say that most of the women I see would be medium to high risk*. *I’d say the majority*. *I’d say probably 80 per cent*, *nearly every second woman that we’re dealing with is high KMMS and has CPFS* [Child Protection and Family Support] *and DV* [domestic violence] issues.

Interview respondent *001*

*When it comes to my clients*, *their perinatal mental health covers a range of factors*, *it can be anything from too much stress at home*, *and that stress is exacerbated by living conditions and access to food*, *levels of alcohol*, *and in particular domestic violence… It is all part of perinatal mental health*.

Interview respondent 006

Many respondents stated that Aboriginal women do not present with complaints of depression and/or anxiety but through a process of exploration and enquiry, women will disclose their stressors and discuss their mental wellbeing.

*No*, *they don’t bring it up in conversation*, *they never say really or bring it up that they’re feeling that way*. *We will sort of have to delve a little bit into the conversation that’s what the KMMS does*.

Interview respondent 001

#### Screening and ongoing support

Healthcare professionals discussed how certain concerns they had around the implementation of the KMMS into routine clinical practice had been resolved through increased and consistent use. These concerns included the time required to complete the KMMS and the skills or confidence to facilitate the psychosocial yarn (Part 2) with patients.

*I would say that timeframe is probably the hardest*, *and you just get more practice with learning how to ask very sensitive questions*, *and I think that’s just with exposure and doing it more frequently*. *I found that was hard initially and then*, *once I got the grasp onto how to actually ask these questions and really find out what I needed to know*, *I found it easier*.

Interview respondent 004

*So generally what happens is the Part 1 I’ll do formally with the woman and I’ll read the questions with her and do it with her*, *and then I’ll invite her up to the bed and do her check on the baby and during the check is when I’ll do Part 2*. *I find they are a lot more relaxed and willing to engage in—sometimes that tough conversation while doing something*, *it is less invasive to them*, *I find*. *From my perspective we don’t have a social worker here at all in* [name of town/community], *so you need to take the time to do it properly*.

Interview respondent 006

Of the 15 online survey respondents seven identified feeling very confident to deliver the KMMS and eight felt somewhat confident. Of the eight that felt somewhat confident respondents discussed lack of experience or discomfort in providing psychosocial care as hampering their confidence:

*I still find it difficult discussing some of the issues involved due to lack of experience in this area of midwifery*.

Survey respondent 012

*I try to integrate topics of discussion into consult*, *rather than asking one by one*. *Sometimes realise later I have forgotten to discuss an issue*.

Survey respondent 005

*Part 2 sections relating to ‘Childhood experiences’ not necessarily feeling confident to deal with issues that may be raised*, *particularly in a population significantly affected by serious trauma*.

Survey respondent 009

Several healthcare professionals noted that many women who disclose stress, trauma or other challenges to their mental health did not wish to pursue formal follow up psychological or counselling-based support. These respondents suggested that the patients in question were satisfied with the yarn and brief intervention provided by them during the KMMS screening process.

*Before we started really understanding how it worked*, *we as a team felt very overwhelmed with all the responsibility of suddenly having these potentially high-risk mental health women who may disclose self-harm and things*. *But the realistic side of things* [doing the KMMS] *just means that women feel that they can get more help out of us by talking and us listening and offering some support*. *But whether some are more willing to go further with the help*, *some say yes and then say no*.

Interview respondent 002

*When using Part 2*, *I know that the woman needs to be given time to discuss challenging issues*, *which can be a barrier in a busy clinic*. *I do feel however that this time is necessary to understand the woman’s situation and build a plan based on her individual needs*. *I also feel that the recommended follow up time frames based on risk can be challenging for a visiting clinician*, *particularly if the woman is only comfortable talking to me*. *I ensure that if this is the case that I document thoroughly and document refusal of referral to other services*.

Survey respondent 008

Healthcare professionals noted the lack of services available to respond to women who require support with housing, food security, and other practical matters correlated to suboptimal perinatal mental health. Some respondents spoke about the local ACCHS Social and Emotional Wellbeing teams as providing a local case management service that was culturally secure and acceptable for women. Other healthcare professionals described patients being reliant on support from visiting services that might cancel due to workforce turnover, community events (such as funerals) or seasonal flooding. All interview respondents identified the need for expanded case management and advocacy services to support women to navigate the complex and multiple challenges they are facing. Respondents also discussed a region wide lack of therapeutic based service provision that promoted recovery and healing.

#### Sample frame—Aboriginal patients

A total of 39 women consented to providing anonymous, written feedback on their experience of the KMMS as a component of their routine perinatal care. The feedback form had five questions, each with predefined response options and an additional free text option. Feedback forms were designed to minimise patient burden and assure anonymity and therefore did not ask for any socio-demographic information. The printed feedback form was provided to the patient by their usual healthcare provider and assistance was available to women to complete the form if the woman requested this support.

#### Asking about perinatal mental health

Nearly all women [38 out of 39] answered ‘yes—important’ when asked ‘do you think it is important that clinics ask about your mood and feelings when you are pregnant or have a young child’. A total of 19 women provided free text responses. These responses centred on the importance of asking the question as a way for clinics to help ‘care for’ and ‘support’ a woman.

*If you hold everything in with no help anything could happen*.

*So at least someone knows how I am going with everything*.

*Take care of me and baby*. *Many people don’t ask*. *Nice to know someone cares*.

*Because there could be things happening at home like overcrowding*, *drinking*.

#### Appropriateness of the KMMS

When asked if the KMMS was the right way for clinics to talk with women about perinatal mental health 29/39 women answered ‘yes—it is the right way’. Nine free text responses were provided. Common themes included the importance of the graphics in KMMS Part 1 helping women to express their feelings. Responses also identified that women may not have anyone else to talk to about their mental health and wellbeing.

*The pictures are a good way*.

*Maybe they don’t have anyone else to talk to and explain their feeling to*.

*Talks right way*.

The remaining 10/39 respondents answered the KMMS was an ‘ok’ way for clinics to discuss perinatal mental health with Aboriginal women. Importantly, no women answered that the KMMS was the ‘wrong way’ to discuss perinatal mental health. Three comments were recorded from participants who responded the KMMS was an ‘ok’ way:

*Some women would like to yarn but it not written down*. *No notes just yarning*.

*Don’t know any other way*.

*This makes it easier to talk*.

#### The structure of the KMMS

When asked if it was important for the KMMS to have the short questions and a yarn (both parts together) 30/39 respondents said ‘both parts together’; six said ‘only the short questions’; three said ‘only the yarn’ and no one responded ‘none of it’.

When asked to explain their answer sixteen additional free text comments were recorded. All of these comments were from participants who responded, ‘both parts together’. Women discussed the importance of yarning so the healthcare professional could understand the woman’s whole story and be best positioned to provide support. Other women described that it was ‘good’ to yarn and helpful for them to reflect on where they were at. The focus on protective factors was also raised as a positive component of the KMMS.

*To know and find out more*. *Words and pictures*. *Good for them to hear what keeps me strong*.

*Feels good how to talk about experiences in childhood*.

*Good to yarn and talk about feelings*. *Help you know how you are feeling*.

*Helps to know the past and know I can be strong now*.

#### KMMS as useful

When asked if ‘doing the KMMS was useful for you’ a total of 33/39 women said yes, four said maybe and two said no. When asked to explain their answer 12 additional comments were recorded. All of these comments were from participants who responded ‘yes’. Respondents discussed how the process of talking felt good, facilitated follow up support and helped develop an understanding between the woman and her healthcare professional

*You get a clear picture*, *you can see me now*.

*These questions never get asked at home*. *Good to open up*.

*Often you don’t stop and think about your feelings*.

*Help me to think more about my life and now getting support and help*.

Women were also asked if the KMMS would be good for members of their family or community. A total of 21 responses were recorded to this question with 20 saying yes and 1 saying no. Four additional comments were recorded. These comments were all from participants who responded ‘yes’. These comments focussed on the importance of talking and the prevalence of psychological distress for Aboriginal people.

Good to talk about stuff

*Lots of family go thru* [sic] *stuff like depression*.

### Domain 3: Ecological system

The results presented in this section represent a desktop analysis of our implementation study project data, specifically how the project engaged, interacted, and informed the delivery of primary healthcare across the Kimberley region.

#### Regional partnerships

The implementation project continued with the same partners and stakeholders who were involved in the KMMS validation study [14], namely the Rural Clinical School of Western Australia (RCSWA), The University of Western Australia; Kimberley Aboriginal Medical Services (KAMS) and its member services (independent regional ACCHSs); and WACHS—Kimberley. The implementation study was funded through a National Health and Medical Research Council Partnership Grant (Grant number 1132659) and the Western Australian Department of Health, with significant in-kind contributions from the partner organisations listed above. The implementation project employed a 0.8 full-time equivalent (FTE) KMMS Project Officer (employed by KAMS; author KF) and a 0.8 FTE KMMS Research Fellow (employed by RCSWA and KAMS; author EC). The WACHS–Kimberley midwife coordinator (author MW) was heavily involved in the project as in-kind support. She attended regular meetings, co-delivered KMMS training to WACHS–Kimberley staff, and was involved in the monitoring and evaluation of the project.

#### KMMS training

The KMMS user manual and the associated KMMS training was revised after the findings of the pre-implementation paper [11]. The revised user manual is publicly available at: https://kahpf.org.au/kmms.

The revised training is in two parts: an overview to perinatal mental health; and KMMS administrator training. The overview to perinatal mental health training was delivered as a 30-minute clinical in-service, the training aimed to build the knowledge of all staff at a clinic location in relation to Aboriginal women and perinatal depression and anxiety. This was a regionally supported approach to awareness raising and capacity building in relation to perinatal mental health.

Administrator training was targeted to health professionals who would be administering the KMMS including Midwives, Child Health Nurses and Aboriginal Health Workers/Practitioners. Many clinic sites chose to send a broader section of their workforce including doctors, Remote Area Nurses, and in some cases administration staff. The training was 60-minutes in duration. It was facilitated by the KMMS Project Officer (KF) and worked through the KMMS training manual. The training provided a context and rationale for the implementation of the KMMS alongside practical activities for staff to build their skills in undertaking the psychosocial yarn, scoring a patient’s risk likelihood and providing a continuum of support for perinatal women.

Details of training delivered were recorded in the KMMS Kimberley training registry, including the number of training episodes, staff members trained and location of the training (Table 3).

**Table 3:** Kimberley Mum’s Mood Scale 2019–2021 training sessions and number of attendees by location.

Training type	Location	Total training sessions delivered	Training session modality: face to face	Training session modality: online	Total training session attendees
Introduction to perinatal mental health	Regional townships	9	8	1	40
	Remote communities	4	4	0	20
Administrator training	Regional townships	56	33	23	130
	Remote community	4	2	2	7
**Total**		**73**	**47**	**26**	**197**

Feedback forms were collected at each training session, with the same form used across both training types. The training feedback forms assessed for increased awareness and knowledge of perinatal mental health issues and support for the implementation of the KMMS. Administration of the KMMS with fidelity to the protocol was assessed via audits reported on in Domain 1: Intervention; and surveys and qualitative interviews with KMMS administrators was reported on in Domain 2: Practice setting.

Most training moved online during 2020 and this continued in 2021, the pivot to online training was a direct result of COVID-19 travel restrictions. A total of 112 completed feedback forms were received over the implementation project.

When asked if the training was ‘interesting and informative’ 103 (92.0%) attendees recorded ‘very’. Similarly, 105 (93.8%) respondents indicated the KMMS training was useful or very useful and 98 (87.5%) respondents stated perinatal mental health was a priority issue for Aboriginal women in the Kimberley. A total of 110 (98.2%) respondents stated they supported the KMMS being implemented into routine clinical care.

Training across the geographically large Kimberley area required sustained and significant engagement. Two case studies are provided to illustrate the human and financial resources required to ensure training occurred.

*Case study 1*: *Remote Community ACCHS*. In order to deliver KMMS training, two phone calls (approximately 10 minutes each), two emails including a clinic requested reschedule of training date (approximately 10 minutes each) and 4 hours of travel on sealed and unsealed roads was recorded. A further three emails (10 minutes each) were exchanged in order to organise a follow up online training session for those staff not able to attend the face-to-face training.

Total non-training time: Approximately 5 hours.

Project staff involved: KMMS Project Officer.

*Case study 2*: *Regional township hospital*. Engaging the service to promote training to staff involved 18 emails (approximately 10 minutes each), two leadership meetings (approximately one hour each involving three KMMS project staff and investigators, and three healthcare provider representatives), two presentations to team meetings (approximately 15 minutes each), six phone calls (average 10 minutes), and three KMMS project team strategy meetings (approximately one hour each involving 4 KMMS project staff and investigators). At the end of the implementation period few staff from this service had attended training.

Total non-training time: Approximately 11.5 hours

Project staff involved: KMMS Project Officer, Research Fellow, two Chief Investigators.

#### Regional stakeholder engagement

Regional stakeholder engagement focussed on providing updates to relevant health networks, committees and forums on the use of the KMMS across the region, seeking advice on ways to increase use, and direct dissemination of academic outputs. Over the implementation period (2018–2021), the KMMS project team recorded 30 separate occasions of regional stakeholder engagement (Table 4).

**Table 4:** Occasion and type of Kimberley stakeholder engagement.

Engagement	Written	Face to face	Total
Kimberley Aboriginal Health Planning Forum (KAHPF)	3	2	**5**
KAHPF Maternal Child Health Subcommittee	1	4	**5**
Kimberley Lead Clinicians Forum	0	7	**7**
KAMS remote services and clinical services team meeting	2	6	**8**
Kimberley Child and Maternal Health annual forum	0	3	**3**
Kimberley Social and Emotional Wellbeing Forum	0	2	**2**
**Total**	**6**	**24**	**30**

#### Informing regional protocols

The KMMS project team were invited to co-lead the development of the Kimberley Perinatal Depression and Anxiety protocol along with select members of the Kimberley Aboriginal Health Planning Forum Maternal Child Health Subcommittee. The protocols form part of clinical inductions for Kimberley health professionals (both ACCHS and WACHS) and are used to ensure best practice approaches are highlighted and consistently applied across the Kimberley. The protocol was developed over a six-month period including 11 face to face meetings and several online draft revisions. The protocol, including a service provider handout, implementation plan and rationale/position paper was endorsed by KAHPF in 2020. The protocol recognises and supports the KMMS as the regionally preferred screening tool for Aboriginal women being screened for perinatal depression and anxiety. The protocol is available at: https://kahpf.org.au/clinical-protocols

#### Sustainability

Ensuring the sustainability of the KMMS was a key consideration over the implementation period. The project team engaged with AMSED eLearning platform to co-design and develop free online KMMS training program (available at https://www.amsed.com.au/kmms-module). The development of this training has enhanced the visibility and accessibility of the KMMS. The training has been widely promoted by health services and relevant peak bodies including Aboriginal Health Council Western Australia, National Aboriginal Community Controlled Health Service, Royal Australian College of GPs. As of February 2022, over 289 individuals have undertaken the KMMS module on AMSED

The project team has worked with individual Kimberley ACCHSs and WACHS to ensure the KMMS forms part of staff inductions. This outcome was a key focus for the KMMS project officer in 2021 and has taken 16 meetings across the region.

In 2019, the KMMS was built into the WACHS electronic medical record system (CHIS). In 2020, the KMMS was built into the ACCHSs electronic medical system (MMEx). The integration of the KMMS into the regional electronic medical systems was a key finding from the pre-implementation paper. The non-integrated system (i.e., asking healthcare professionals to scan paper-based assessments into a patient files) acted as a deterrent to both KMMS use and good clinical governance with scanned KMMS paperwork filed inconsistently in patients’ electronic medical records. In 2021, the project team were approached by Communicare (another large electronic medical record system in Australia). Communicare asked for permission to build KMMS into their system as many ACCHSs and women’s health services across Australia had requested its inclusion. As of December 2021, the KMMS is available to over 250 organisations across Australia.

We have worked with the key peak bodies such as the Centre of Perinatal Excellence to ensure the KMMS is captured in the Australian Clinical Guidelines to further embed sustainability and support broader uptake of the KMMS.

## Discussion

There was a 1.5-fold increase in overall recorded perinatal mental health screening (KMMS and EPDS) and a 3.5-fold increase in recorded KMMS screening of Aboriginal women who attended an antenatal appointment at a Kimberley ACCHS in T2 compared to T1. Fidelity to the tool, using both parts together and recording an overall risk assessment as per training guidelines also greatly improved (from 2.3% to 61.8%). This improved fidelity enabled the identification of 23% of women screened in T2 with at least one elevated risk assessment. These findings, which are similar to the original KMMS validation study [14], highlight the amplified risk faced by Aboriginal women in relation to perinatal mental ill health [5,31].

The acceptability of providing mental health screening and support to patients from the perspective of healthcare professionals has returned divergent results. A recent review found that midwives are increasingly aware of the impacts of perinatal mental health and are interested in routinely providing mental health care and support [32]. Other research has found that healthcare professionals (including midwives) can be uncomfortable providing mental health screening and psychosocial care [33,34]. Our pre implementation study [11] found Kimberley healthcare professionals were similarly ambivalent about administering a psychosocial screening tool. For Kimberley healthcare professionals involved in this study it was identified that regular use of the KMMS helped to mitigate perceived barriers such as not having the skills or time required to undertake the psychosocial yarn and provide the subsequent psychosocial care. This finding is congruent with other studies that suggest many healthcare professionals lack confidence in facilitating psychosocial screening with patients [32,34–36] but with training, support, and consistency of use, healthcare professionals gain the confidence to provide psychosocial screening and care [37,38].

The limited role of Aboriginal staff in administering the KMMS was noted during the study. Previous studies have suggested that Aboriginal women would find KMMS screening by Aboriginal staff acceptable and in some cases preferable to non-Aboriginal staff [10,11]. An evaluation of the Apunipima Cape York Health Council, Baby One Program has demonstrated the positive impacts of having designated Aboriginal staff to undertake mental health screening and provide psychosocial support inclusive of case management as required for perinatal patients [39]. Additional strategies are needed to increase the role of Aboriginal Health Workers/Practitioners in the provision of mental health care including screening and support [40]. An important step would be nesting designated Aboriginal professionals within Maternal and Child Health teams.

Observed through the implementation study is how all components of the Dynamic Sustainability Framework are necessary for implementation success. The co-designed and resiliency inclusive ‘intervention’ continued to demonstrate user acceptability within the practice setting (Aboriginal women patients and healthcare providers). At the ecological system level the partnership and investment by the Kimberley ACCHSs, WACHS–Kimberley and RCSWA has been foundational to the positive implementation traction [41]. Of particular significance is the endorsement of the KMMS by the Kimberley Aboriginal Health Planning Forum as the preferred perinatal depression and anxiety screening tool for Kimberley Aboriginal women [42]. This endorsement has provided legitimacy for the KMMS implementation team to work with clinics throughout the region. Specifically, it has fostered access to healthcare professionals for KMMS training purposes.

A strength of this study is that it reports on the real world implementation of a mental health screening initiative in an environment characterised by low mental health resources [6] and high workforce turn over [43]. A benefit of the real-world, real-time nature of the study is that healthcare professionals who participated in the study were supported to use the KMMS and in turn we saw healthcare professionals increasingly administer the KMMS with fidelity to the model. This study was undertaken by an Aboriginal and non-Aboriginal team and demonstrates that implementation of good clinical practice is possible with a sustained multi-year investment, strong partnerships, and a regionally embedded implementation team. While the audit of Kimberley ACCHS was comprehensive, the absence of WACHS–Kimberley implementation data limits the quantitative assessment of KMMS use to the Kimberley ACCHS.

Another limitation is that this study did not interview Aboriginal women about their experience of the KMMS in the same way that we interviewed healthcare providers. The decision to rely solely on feedback forms from Aboriginal women who had been screened using the KMMS was made in respect of participant research burden [22] and the complication of COVID-19 travel restrictions which prevented access by researchers to remote communities. We are grateful that many women chose to provide free text responses on the KMMS feedback form, these responses allowed us to assess key themes and provide a richer qualitative descriptive understanding of acceptability. While outside of our control we are cognisant of selection bias in healthcare professionals that chose to be interviewed and to a lesser extent complete the online surveys [44]. The healthcare professionals who completed the qualitative interviews can be understood as champions of the KMMS, however their stories are valid and integral to the implementation of the tool across the region. It is noteworthy that several of these respondents became champions during the implementation period despite initial pre-implementation hesitancy.

## Conclusion

Improving perinatal mental health screening practices for Aboriginal women is an issue of national significance. This study has demonstrated the real-world ability of the KMMS to improve screening practices for Aboriginal women in the Kimberley. Across the Dynamic Sustainability Framework domains of intervention, practice setting, and ecological systems a positive implementation trajectory was achieved for the KMMS. The resources and partnerships required to translate this research project into routine clinical care were significant and demonstrate the need for research implementation funding to be a sustained multi-year undertaking. This includes dedicated funded positions to progress the widely acknowledged but poorly articulated work of implementation. Further work is required across the Kimberley health services to support Aboriginal staff to be involved in the screening and care of Aboriginal women’s perinatal mental health.

## Supporting information

S1 Checklist Statement against Standards for Reporting Implementation Studies (StaRI). (DOCX)

S2 Checklist PLOS ONE inclusivity checklist. (DOCX)

S1 File Survey questions and qualitative interview guide. (DOCX)

## References

[1] Austin M P, Highet N. Mental health care in the perinatal period: Australian Clinical Practice Guideline Melbourne: Centre of Perinatal Excellence; 2017 [updated 3 July 2019. https://www.cope.org.au/health-professionals/health-professionals-3/review-of-new-perinatal-mental-health-guidelines/.

[2] Cox JL, Holden JM, Sagovsky R. Detection of postnatal depression. Development of the 10-item Edinburgh Postnatal Depression Scale. Br J Psychiatry. 1987;150:782–6. doi: 10.1192/bjp.150.6.782 3651732

[3] San Martin Porter MA, Betts K, Kisely S, Pecoraro G, Alati R. Screening for perinatal depression and predictors of underscreening: findings of the Born in Queensland study. Med J Aust. 2019;210(1):32–7. doi: 10.5694/mja2.12030 30636310

[4] Zubrick SR, Dudgeon P, Gee G, Glaskin B, Kelly K, Paradies Y, et al. Social determinants of Aboriginal and Torres Strait Islander social and emotional wellbeing. In: Dudgeon P, Milroy H, Walker R, editors. Working Together: Aboriginal and Torres Strait Islander Mental Health and Wellbeing Principles and Practice. Canberra: Australian Government Department of the Prime Minister and Cabinet; 2004.

[5] Gausia K, Thompson SC, Nagel T, Schierhout G, Matthews V, Bailie R. Risk of antenatal psychosocial distress in indigenous women and its management at primary health care centres in Australia. Gen Hosp Psychiatry. 2015;37(4):335–9. doi: 10.1016/j.genhosppsych.2015.04.005 25920681

[6] Bhat SK, Marriott R, Galbally M, Shepherd CC. Psychosocial disadvantage and residential remoteness is associated with Aboriginal women’s mental health prior to childbirth. International Journal of Population Data Science. 2020;5(1). doi: 10.23889/ijpds.v5i1.1153 32935056 PMC7473279

[7] Kotz J, Marriott R, Reid C. The EPDS and Australian Indigenous women: A systematic review of the literature. Women Birth. 2021;34(2):e128–e34. doi: 10.1016/j.wombi.2020.02.007 32144025

[8] Hughes K, Bellis MA, Hardcastle KA, Sethi D, Butchart A, Mikton C, et al. The effect of multiple adverse childhood experiences on health: a systematic review and meta-analysis. Lancet Pub Health. 2017;2(8):e356–e66. doi: 10.1016/S2468-2667(17)30118-4 29253477

[9] Kotz J, Munns A, Marriott R, Marley JV. Perinatal depression and screening among Aboriginal Australians in the Kimberley. Contemp Nurse. 2016;52(1):42–58. doi: 10.1080/10376178.2016.1198710 27294330

[10] Carlin E, Atkinson D, Marley JV. ‘Having a quiet word’: yarning with Aboriginal women in the Pilbara region of Western Australia about mental health and mental health screening during the perinatal period. Int J Environ Res Public Health. 2019;16(21):4253. doi: 10.3390/ijerph16214253 31683908 PMC6862568

[11] Carlin E, Spry E, Atkinson D, Marley JV. Why validation is not enough: Setting the scene for the implementation of the Kimberley Mum’s Mood Scale. PLoS One. 2020;15(6):e0234346. doi: 10.1371/journal.pone.0234346 32530934 PMC7292413

[12] Lin I, Green C, Bessarab D. ’Yarn with me’: applying clinical yarning to improve clinician-patient communication in Aboriginal health care. Aust J Prim Health. 2016;22(5):377–82. doi: 10.1071/PY16051 28442021

[13] Carlin E, Ferrari K, Seear K, Spry E, Williams M, Engelke C, et al. Kimberley Mum’s Mood Scale (KMMS) Training Manual. Broome, Western Australia; 2019 July 2019.

[14] Marley JV, Kotz J, Engelke C, Williams M, Stephen D, Coutinho S, et al. Validity and Acceptability of Kimberley Mum’s Mood Scale to Screen for Perinatal Anxiety and Depression in Remote Aboriginal Health Care Settings. PLoS One. 2017;12(1):e0168969. doi: 10.1371/journal.pone.0168969 28135275 PMC5279756

[15] Chambers DA, Glasgow RE, Stange KC. The dynamic sustainability framework: addressing the paradox of sustainment amid ongoing change. Implement Sci. 2013;8(1):117. doi: 10.1186/1748-5908-8-117 24088228 PMC3852739

[16] Damschroder LJ, Aron DC, Keith RE, Kirsh SR, Alexander JA, Lowery JC. Fostering implementation of health services research findings into practice: a consolidated framework for advancing implementation science. Implement Sci. 2009;4:50. doi: 10.1186/1748-5908-4-50 19664226 PMC2736161

[17] Pinnock H, Barwick M, Carpenter CR, Eldridge S, Grandes G, Griffiths CJ, et al. Standards for Reporting Implementation Studies (StaRI) Statement. BMJ. 2017;356:i6795. doi: 10.1136/bmj.i6795 28264797 PMC5421438

[18] Anguera MT, Blanco-Villaseñor A, Losada JL, Sánchez-Algarra P, Onwuegbuzie AJ. Revisiting the difference between mixed methods and multimethods: Is it all in the name? Quality & Quantity. 2018;52(6):2757–70.

[19] Sekhon M, Cartwright M, Francis JJ. Acceptability of healthcare interventions: an overview of reviews and development of a theoretical framework. BMC Health Serv Res. 2017;17(1):88. doi: 10.1186/s12913-017-2031-8 28126032 PMC5267473

[20] El-Den S, O’Reilly CL, Chen TF. A systematic review on the acceptability of perinatal depression screening. J Affect Disord. 2015;188:284–303. doi: 10.1016/j.jad.2015.06.015 26386439

[21] Bradshaw C, Atkinson S, Doody O. Employing a Qualitative Description Approach in Health Care Research. Glob Qual Nurs Res. 2017;4:1–8. doi: 10.1177/2333393617742282 29204457 PMC5703087

[22] National Health and Medical Research Council. Values and Ethics—Guidelines for Ethical Conduct in Aboriginal and Torres Strait Islander Health Research. Canberra: Commonwealth of Australia; 2018.

[23] WA Country Health Services Planning and Evaluation Unit. Kimberley Health Profile. http://www.wacountry.health.wa.gov.au/fileadmin/sections/publications/Publications_by_topic_type/Reports_and_Profiles/eDoc_-_CO_-_Kimberley_Health_Profile_2018.pdf (accessed 30 Jan 2021).

[24] Australian Bureau of Statistics. 2016 QuickStats Kimberley.

[25] Australian Institute of Health and Welfare (AIHW). Aboriginal and Torres Strait Islander Health Performance Framework 2020 summary report. Cat. no. IHPF 2. Canberra, Australia: AIHW; 2020.

[26] McPhee R, Carlin E, Seear K, Carrington-Jones P, Sheil B, Lawrence D, et al. Unacceptably high: an audit of Kimberley self-harm data 2014–2018. Australasian Psychiatry. 2021:10398562211010790. doi: 10.1177/10398562211010790 33951955 PMC8894678

[27] Hutchinson M, Joyce A, Peirce A. Western Australia’s Mothers and Babies, 2015: 33rd Annual Report of the Western Australian Midwives’ Notification System,. Perth, Western Australia: Department of Health, Western Australia; 2019.

[28] Hsieh H-F, Shannon SE. Three Approaches to Qualitative Content Analysis. Qual health research. 2005;15(9):1277–88. doi: 10.1177/1049732305276687 16204405

[29] Dudgeon P, Boe M, Walker R. Addressing inequities in Indigenous mental health and wellbeing through transformative and decolonising research and practice. Journal of Research in Health Science. 2020;5(3).

[30] National Health and Medical Research Council (NHMRC). Ethical conduct in research with Aboriginal and Torres Strait Islander Peoples and communities: Guidelines for researchers and stakeholders. https://www.nhmrc.gov.au/about-us/resources/ethical-conduct-research-aboriginal-and-torres-strait-islander-peoples-and-communities (accessed 7 Aug 2021). Commonwealth of Australia: Canberra 2018.

[31] Bowen A, Duncan V, Peacock S, Bowen R, Schwartz L, Campbell D, et al. Mood and anxiety problems in perinatal Indigenous women in Australia, New Zealand, Canada, and the United States: A critical review of the literature. Transcultural psychiatry. 2014;51(1):93–111. doi: 10.1177/1363461513501712 24065605

[32] Coates D, Foureur M. The role and competence of midwives in supporting women with mental health concerns during the perinatal period: A scoping review. Health & social care in the community. 2019;27(4):e389–e405. doi: 10.1111/hsc.12740 30900371

[33] Mellor A C, Payne C D, McAra-Couper C J. Midwives’ perspectives of maternal mental health assessment and screening for risk during pregnancy. New Zealand College of Midwives Journal. 2019;55:27–34.

[34] Viveiros CJ, Darling EK. Perceptions of barriers to accessing perinatal mental health care in midwifery: a scoping review. Midwifery. 2019;70:106–18. doi: 10.1016/j.midw.2018.11.011 30611114

[35] Hauck YL, Kelly G, Dragovic M, Butt J, Whittaker P, Badcock JC. Australian midwives knowledge, attitude and perceived learning needs around perinatal mental health. Midwifery. 2015;31(1):247–55. doi: 10.1016/j.midw.2014.09.002 25262025

[36] Jones CJ, Creedy DK, Gamble JA. Australian midwives’ attitudes towards care for women with emotional distress. Midwifery. 2012;28(2):216–21. doi: 10.1016/j.midw.2010.12.008 21342738

[37] Price SK, Corder-Mabe J, Austin K. Perinatal depression screening and intervention: enhancing health provider involvement. J Womens Health. 2012;21(4):447–55. doi: 10.1089/jwh.2011.3172 22309209 PMC3321675

[38] Schmied V, Reilly N, Black E, Kingston D, Talcevska K, Mule V, et al. Opening the door: midwives’ perceptions of two models of psychosocial assessment in pregnancy- a mixed methods study. BMC Pregnancy Childbirth. 2020;20(1):451. doi: 10.1186/s12884-020-03133-1 32767969 PMC7412833

[39] Campbell S, McCalman J, Redman-MacLaren M, Canuto K, Vine K, Sewter J, et al. Implementing the Baby One Program: a qualitative evaluation of family-centred child health promotion in remote Australian Aboriginal communities. BMC Pregnancy Childbirth. 2018;18(1):73. doi: 10.1186/s12884-018-1711-7 29573747 PMC5866524

[40] O’Keefe VM, Cwik MF, Haroz EE, Barlow A. Increasing culturally responsive care and mental health equity with indigenous community mental health workers. Psychol Serv. 2019. doi: 10.1037/ser0000358 31045405 PMC6824928

[41] Kellam SG. Developing and maintaining partnerships as the foundation of implementation and implementation science: reflections over a half century. Administration and Policy in Mental Health and Mental Health Services Research. 2012;39(4):317–20. doi: 10.1007/s10488-011-0402-8 22240938 PMC4422186

[42] Kimberley Aboriginal Health Planning Forum. Perinatal Depression and Anxiety Protocol https://kahpf.org.au/s/Perinatal-Depression-and-Anxiety.pdf2019 [01/03/2019].

[43] Wakerman J, Humphreys J, Russell D, Guthridge S, Bourke L, Dunbar T, et al. Remote health workforce turnover and retention: what are the policy and practice priorities? Hum Resourc Health. 2019;17(1):99. doi: 10.1186/s12960-019-0432-y 31842946 PMC6915930

[44] Emanuel EJ, Wendler D, Grady C. What Makes Clinical Research Ethical? JAMA. 2000;283(20):2701–11. doi: 10.1001/jama.283.20.2701 10819955

