# Supplementary material for: Implementation of the ‘Kimberley Mum’s Mood Scale’ across primary health care services in the Kimberley region of Western Australia: A mixed methods assessment
Source: PLoS One. 2022 Sep 2;17(9):e0273689. doi: 10.1371/journal.pone.0273689 (PMC9439224; doi:10.1371/journal.pone.0273689)
Supplement: S1 File — (DOCX) [file pone.0273689.s003.docx]

# Health Professional In-Depth Interview: Use and Perceptions of the KMMS

**Opening Statement**

Hello, my name is . <Describe your position and background>, Thank you for taking the time today to speak to me.

You have been asked to participate in this interview as a health professional who has used the KMMS to screen Aboriginal mums. We would like to hear from you about your experience with the KMMS.

During the interview I will ask you some questions about your experience using the KMMS. Please keep in mind that there are no ‘right’ or ‘wrong’ answers to any of the questions I will ask. The purpose is to seek your opinions on the KMMS. Please note that while we talk I will be writing notes and/or audio recording. Please let me know if you are comfortable for me to audio record. These notes and the comments you make will remain confidential and your name will not be attached to any comments you make.

The interview should take about 30 minutes to 1 hour. Do you have any questions before we begin?

# Opening Question

Q1: *How long have you been working for a Health Service in the Kimberley and what is your current role?*

# Transition Questions

Q2: *Is perinatal mental health an issue you come across regularly with your patients?*

PROBE: What are the signs and symptoms you associate with PNDA?

PROBE: Are women familiar with the terms depression and anxiety?

# Key Questions

Q3: *Do you use the KMMS to screen for depression and anxiety during the perinatal period? Can you explain this further?*

PROBE: How many times have you used the KMMS; 0-10; 10-20; 20 plus PROBE: What do you see as the benefits of the KMMS?

PROBE: Does the language, format, length/time, or issues with the venue or other factors constrain/ enhance use of the KMMS? Can you explain this further?

PROBE: Are there times when you have used the Edinburgh Perinatal Depression Screening tool instead of the KMMS? Can you explain the reasons to me?

PROBE: Do you associate any challenges with using the KMMS? PROBE: Do you feel confident using the KMMS?

Q4: *Please describe how you use the KMMS with patients?*

PROBE: How often do you use the KMMS during the perinatal period? Are you using it according to the EPDS schedule, more or less? Please tell me a bit about your answer.

PROBE: Do you use the KMMS when a woman’s spouse or other family members are present or just with the patient by herself? Can you explain the reasons to me?

PROBE: Please describe how the patient is referred for further care if they score as Moderate or High Risk of depression and anxiety?

PROBE: Please describe how you monitor the KMMS support/ follow up actions with the patient?

Q5: *How have your patients responded to the KMMS?*

PROBE: Can you tell me about how your patients have responded to Part 1/Part 2 of the KMMS?

PROBE: Do you think the KMMS has helped your patient’s understanding and management of their perinatal mental health? Can you explain this further?

Q6: *How useful do you think the KMMS is?*

PROBE: Are you supportive of the KMMS being implemented as standard practise across the region? Can you explain the reasons for this?

PROBE: Has the KMMS changed your approach to thinking, talking, managing or supporting Aboriginal women’s perinatal mental health?

Q7: *What improvements or changes would you make to the KMMS or how it is used?*

# Closing questions

Q8: *Of all the issues discussed today (facilitator to summarise briefly), have we missed anything*

# Health Professional Survey Questions (administered via survey monkey)

1. Please list your job title

2. Have you been trained to use the KMMS? (If no, please do not continue the survey)

Yes/ No

3. How many times have you used the KMMS in the last 6 months?

0/ 1-5/ 6-10/ 11+

Comments

4. In your experience, is the KMMS the most appropriate perinatal mental health screening tool for Kimberley Aboriginal women?

Yes/ No

Comments

5. In the last 6 months have you used the EPDS instead of the KMMS when screening Aboriginal women?

Yes, I use the EPDS all the time

Yes, I use the EPDS sometimes

No, I only used the KMMS

Comments

6. From your experience are there benefits of using the KMMS

Many benefits

Some benefits

No benefits

I am not using the KMMS

Comments

7. Have you experienced any barriers or challenges when you are using the KMMS?

Many challenges

Some challenges

No challenges

I am not using the KMMS

Comments

8. How confident do you feel using the KMMS?

Very confident

Somewhat confident

Not confident

I am not using the KMMS

Comments

9. Was the training you received sufficient for you to use the KMMS?

Yes

No

Comments

10. How are you using the KMMS?

In accordance with the screening schedule (twice during pregnancy and three times in the first postnatal year)

As an ongoing approach (regular check-ins)

Not using the KMMS

Comments
